# Long-Term outcomes of uncut roux-en-Y anastomosis in laparoscopic distal gastrectomy: A retrospective analysis

**DOI:** 10.3389/fsurg.2023.1090626

**Published:** 2023-02-22

**Authors:** Guangxu Zhu, Shengjie Zhou, Xiaoru Shen, Jianjun Qu

**Affiliations:** ^1^ Department of General Surgery, Weifang People’s Hospital, Weifang, China; ^2^ Department of Anesthesiology, Weifang People’s Hospital, Weifang, China; ^3^ Department of General Surgery, Chengdu Fifth People’s Hospital, Chengdu, China

**Keywords:** gastric cancer, laparoscopic surgery, uncut Roux-en-Y anastomosis, billrothII+Braun anastomosis, quality of life

## Abstract

**Background:**

Uncut Roux-en-Y (U-RY) has been increasingly used in radical gastric cancer surgery, but it is still in the exploratory stage. There is insufficient evidence for its long-term efficacy.

**Methods:**

From January 2012 to October 2017, a total of 280 patients diagnosed with gastric cancer were eventually included in this study. Patients undergoing U-RY were assigned to the U-RY group, while patients undergoing BillrothII with Braun (B II + Braun) were assigned to the B II + Braun group.

**Results:**

There were no significant differences between the two groups in operative time, intraoperative blood loss, postoperative complications, first exhaust time, time to liquid diet, and length of postoperative hospital stay (all *P* > 0.05). Endoscopic evaluation was performed 1 year after surgery. Compared to B II + Braun group, the uncut Roux-en-Y group had significantly lower incidences of gastric stasis [16.3% (15/92) vs. 28.2% (42/149), *χ*^2^ = 4.448, *P* = 0.035], gastritis [13.0% (12/92) vs. 24.8% (37/149), *χ*^2^ = 4.880, *P* = 0.027] and bile reflux [2.2% (2/92) vs. 20.8% (11/149), *χ*^2^ = 16.707, *P* < 0.001], and the differences were statistically significant. The questionnaire was completed 1 year after surgery,the QLQ-STO22 scores showed that, the uncut Roux-en-Y group had a lower pain score(8.5 ± 11.1 vs. 11.9 ± 9.7, *P* = 0.009) and reflux score(7.9 ± 8.5 vs. 11.0 ± 11.5, *P* = 0.012), and the differences were statistically significant (*P* < 0.05). However, there was no significant difference in overall survival (*P* = 0.688) and disease-free survival (*P* = 0.505) between the two groups.

**Conclusion:**

Uncut Roux-en-Y has the advantages of better safety, better quality of life and fewer complications, and is expected to be one of the best methods for digestive tract reconstruction.

## 1. Introduction

Gastric cancer is one of the most common malignancies in the digestive system, and radical gastrectomy is the most effective treatment (1). There are few studies on the long-term outcomes of laparoscopic U-RY digestive tract reconstruction after resection of distal gastric cancer. Domestic and foreign experts still have great controversy about the advantages and disadvantages of U-RY digestive tract reconstruction after laparoscopic resection (2, 3).

In this study, we aimed to explore the long-term outcomes of uncut Roux-en-Y anastomosis in radical laparoscopic distal gastrectomy.

## 2. Methods

### 2.1. Selection of enrolled patients

**Figure 1:**
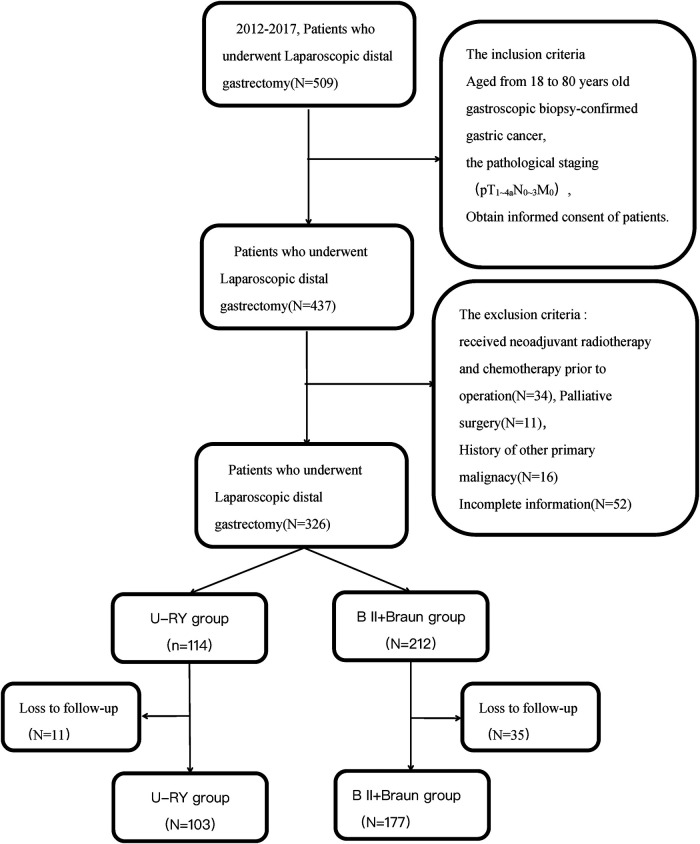
Flowchart of the study, 280 patients were included in this study.

The study was conducted in accordance with the Helsinki Declaration (as revised in 2013). This study was approved by the Ethics Committee of Weifang People's Hospital. All patients consented to data being used for research when receiving treatment. Inclusion criteria were as follows: Aged 18 to 80, gastroscopic biopsy-confirmed gastric cancer, pathological staging (pT1∼4_a_N0∼3M0), obtain informed consent of patients. Exclusion criteria were as follows:received neoadjuvant radiotherapy and chemotherapy prior to surgery, Palliative surgery, history of other primary malignacies, incomplete information or loss to follow-up. A flowchart of the study is shown in Figure 1.

The study protocol was approved by the research ethics committee of Weifang People's Hospital. Written informed consent was obtained from all patients prior to surgery. Each patient signed an informed consent to allow their treatment-related information to be used in future studies.

### 2.2. Surgical procedure

All patients underwent laparoscopic distal gastrectomy with D2 lymphadenectomy based on Japanese gastric cancer treatment guidelines. Digestive tract reconstruction was performed *in vitro*.

**Figure 2:**
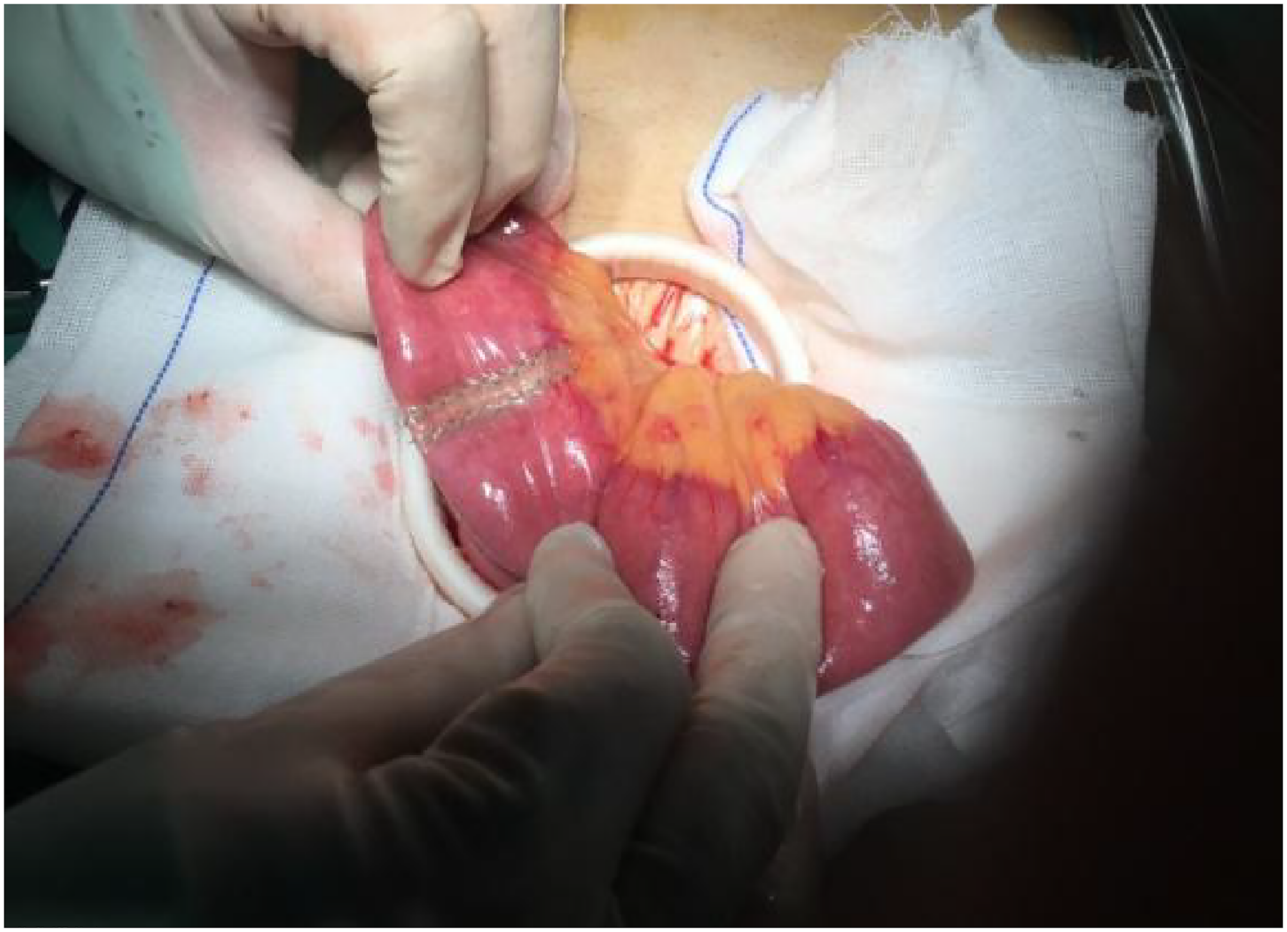
Adopt four-row staplers (knifeless) methods of jejunal occlusion.

**Figure 3:**
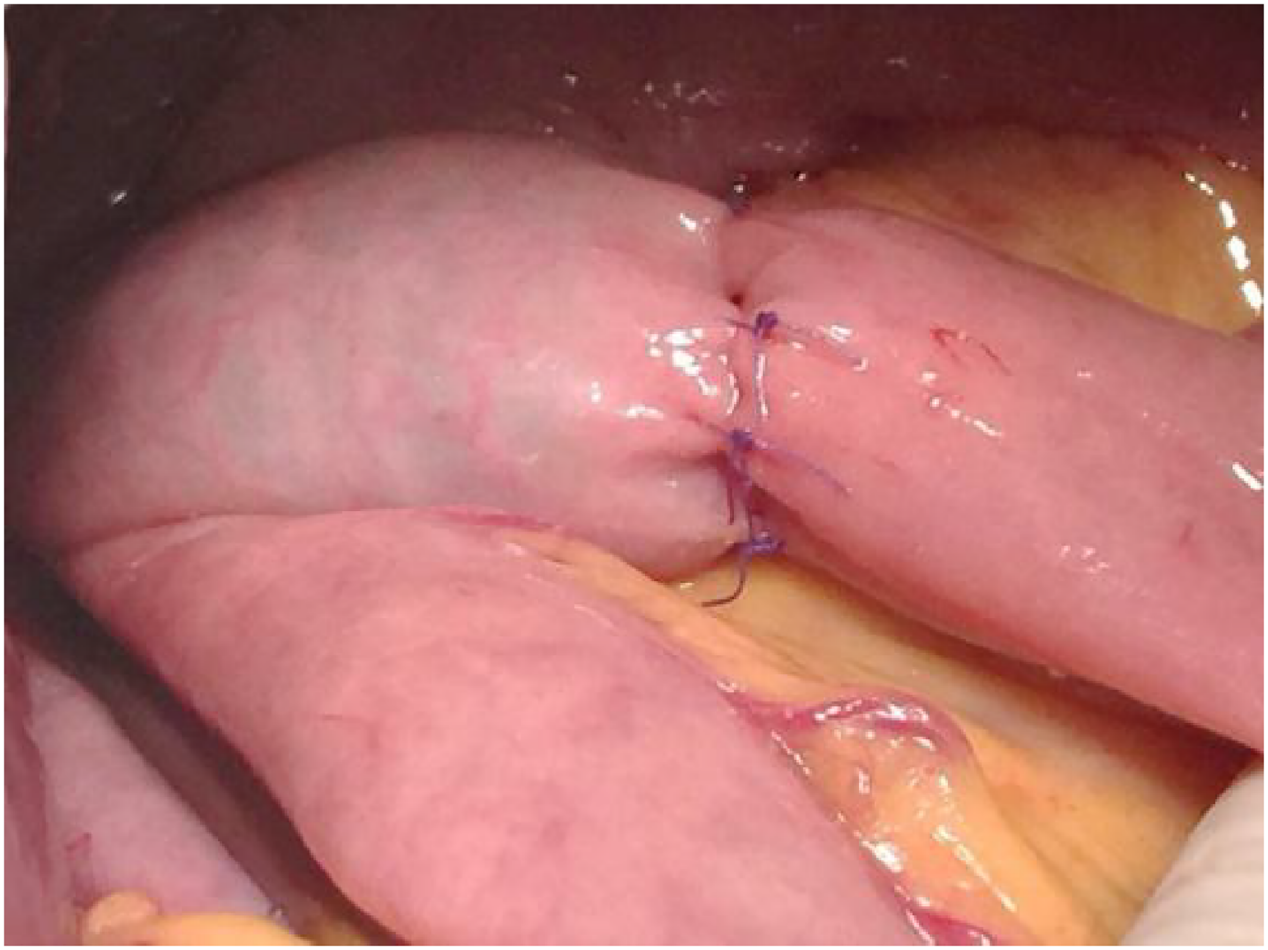
Reinforced by interrupted full-thickness sutures at the occlusion site.

U-RY and B II + Braun reconstructions were established using mechanical staples. In the U-RY group, gastrojejunal anastomosis was established between the residual stomach and jejunum 25 cm distal to Treitz ligament after distal gastrectomy. Then, a side-to-side jejunojejunostomy was established between the afferent and efferent jejunal limbs approximately 30 cm away from the site of gastrojejunal anastomosis and 25 cm away from Treitz ligament. Finally, the jejunal lumen was occluded using the four-row (knifeless) stapler method at a site 3–5 cm proximal to gastrojejunal anastomosis (Figures 2, 3). Compared to U-RY group, B II + Braun group lacked the steps of the jejunal lumen that occluded, and other surgical procedures were the same.

**Figure 4:**
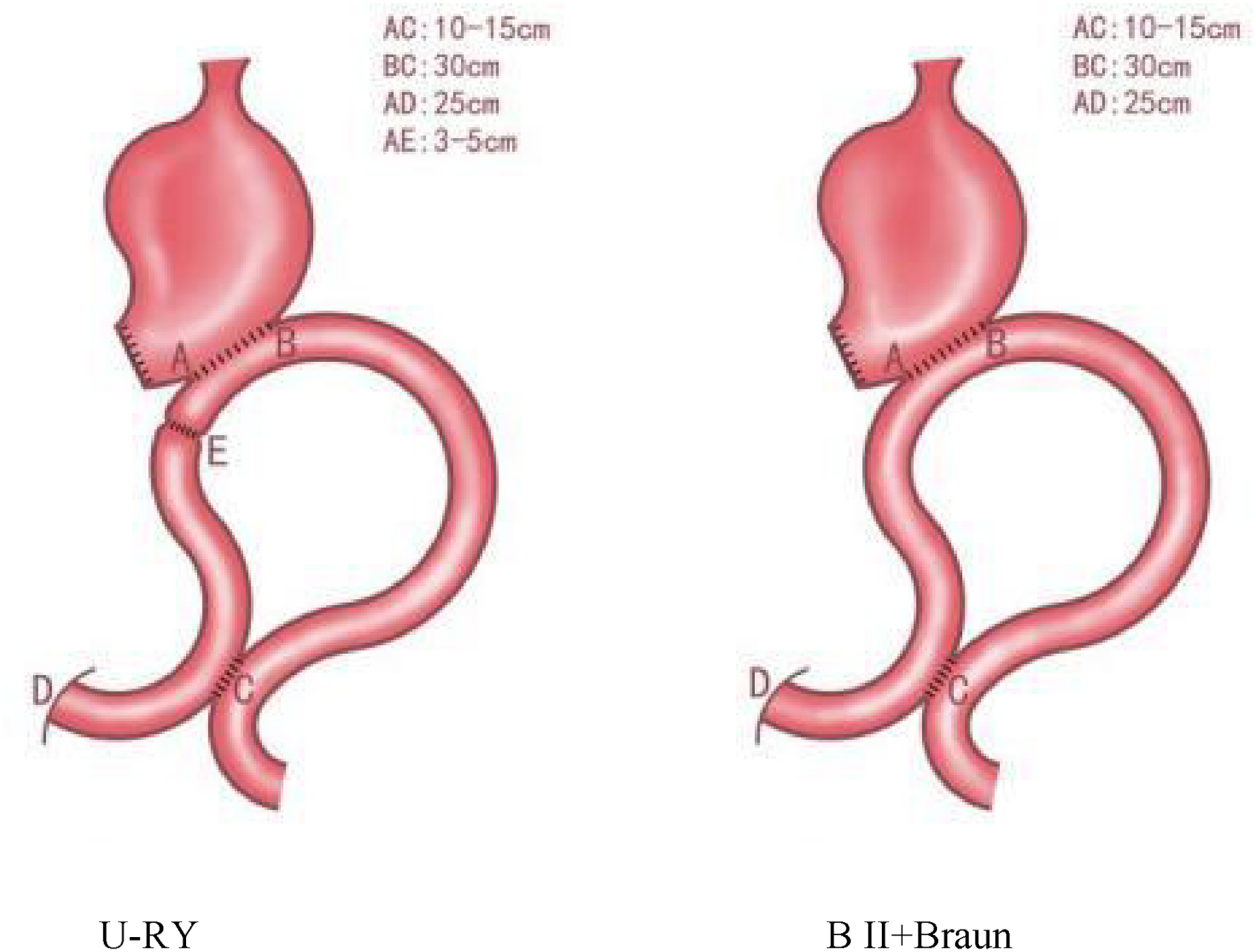
The two methods of digestive tract reconstruction are depicted.

All operations on patients with gastric cancer were performed by the same surgical team. Both methods of reconstructing the digestive tract are described in Figure 4.

### 2.3. Data collection and follow-up

The Operation time, digestive tract reconstruction time, intraoperative blood loss, first exhaust time, postoperative hospital stay, and perioperative complications were monitored during the perioperative period. The QoL index of the two groups 1 year after surgery was assessed using the QLQ-STO22 questionnaires. The QLQ-STO22 responses were linearly converted into scores ranging from 0 to 100 according to the EORTC scoring manual. High scores for items related to global health status and functions and low scores for items related to symptoms and single items (i.e., Appetite loss, Dyspnea, dry mouth, taste, body image, and hair loss) indicate a favorable QoL (4). Postoperative QoL scores were collected 1 year after surgery by telephone calls, letters, or outpatient visits. The follow-up period was until June 2022.

Patients were followed up every 3 months at the first 2 years after surgery and every 6 months at the following 3 years until the patient died or the study was terminated. Patients were followed up by outpatient appointment, letter or telephone consultation. The median follow-up was 46 months in the U-RY group and 46 months in the B II + Braun group. All patients were followed until death or the end of the study in June 2022.

### 2.4. Statistical analysis

SPSS version 26.0 software (SPSS, Chicago, IL, USA) was used for statistical analysis. Continuous variables were compared using the Student's *t*-test and are expressed as mean ± standard deviation. The frequencies of categorical variables are expressed as rates, and rates are compared using the chi-squared test or Fisher's exact test. Here, *P* < 0.05 was considered

statistically significant.

## 3. Results

Finally, 280 patients (male = 226, 80.7%; female = 54, 19.3%) were included in this study. These patients were divided into the U-RY group (*n* = 103) and B II + Braun group (*n* = 177) according to the technique used for digestive tract reconstruction. Baseline characteristics of the two groups were comparable (Table 1).

**Table 1:** Baseline information for two groups of patients.

	U-RY group (*n* = 103)	B II + Braun group (*n* = 177)	T/*χ*^2^	*P*-value
Sex, *N* (%)			0.02	0.966
Male	83 (80.58)	143 (80.79)		
Female	20 (19.42)	34 (19.21)		
BMI, *N* (%)			1.330	0.722
<18.5 (thin)	2 (1.94)	4 (2.26)		
18.5–25 (normal)	71 (68.93)	114 (64.41)		
25–30 (over weight)	27 (35.92)	56 (31.64)		
≥30 (obesity)	3 (2.91)	3 (1.69)		
ASA grade, *N* (%)			0.902	0.342
I/II	86 (63.50)	155 (87.57)		
III	17 (16.50)	22 (12.43)		
Tumor size, *N* (%)			0.032	0.859
<4 cm	35 (33.98)	62 (35.03)		
≥4 cm	68 (66.02)	115 (64.97)		
Preoperative CEA, *N* (%)			0.002	0.967
Positive	84 (81.55)	144 (81.36)		
Negative	19 (18.45)	33 (18.64)		
Pathologic T stage, *N* (%)			0.891	0.827
T1	17 (16.50)	26 (14.69)		
T2	31 (30.10)	55 (31.07)		
T3	43 (41.75)	69 (38.98)		
T4a	12 (11.65)	27 (15.25)		
Pathologic *N* stage, *N* (%)			5.205	0.267
N0	3 (2.91)	11 (6.21)		
N1	43 (41.75)	73 (41.24)		
N2	31 (30.10)	61 (79.22)		
N3	26 (25.24)	30 (16.95)		
Pathologic TNM stage, *N* (%)			1.194	0.754
I	12 (11.65)	24 (13.56)		
II	49 (47.57)	73 (41.24)		
III	51 (49.51)	79 (44.63)		

No deaths were recorded during the perioperative period. There were no significant differences between the two groups in operative time, intraoperative blood loss, postoperative complications, time to flat, time to liquid diet, and length of postoperative hospital stay (all *P* > 0.05). The incidence of specific complications in the two groups were 15.53% (16/103) and 14.12% (25/177), respectively, but the difference was not statistically significant. In the BII + Braun group, one patient developed duodenal stump anastomotic fistula after surgery and was discharged with improvement after a second operation (Table 2).

**Table 2:** Comparison of postoperative conditions between the two groups.

	U-RY group (*n* = 103)	BII + Braun group (*n* = 177)	T/χ^2^	*P*-value
Operative time (min)	237.6 ± 26.9	231.8 ± 22.9	1.928	0.055
Estimated blood loss, (ml)	85.8 ± 11.9	89.6 ± 18.1	1.904	0.058
Harvested lymph nodes (*n*)	24.1 ± 5.5	25.9 ± 9.5	1.817	0.070
Mean gas passing, (days)	68.6 ± 10.4	70.7 ± 12.7	1.466	0.144
Mean postoperative hospital stay, (days)	11.7 ± 3.7	12.0 ± 2.7	0.896	0.371
Histology, *N* (%)			1.071	0.585
Differentiated	64 (62.14)	99 (55.93)		
Undifferentiated	35 (33.98)	69 (38.98)		
Other	4 (3.88)	9 (5.08)		
Postoperative complication, *N* (%)	16 (15.53)	25 (14.12)	0.104	0.748
Anastomotic leakage	2	3		
anastomotic stenosis	1	0		
Intra-abdominal infection	3	5		
Abdominal bleeding	1	2		
Ileus	0	2		
Pneumonia	4	7		
Incision-related complications	1	3		
Duodenal stump fistula	0	1		
Delayed gastric emptying	2	2		
Clavien-Dindo complication grade, *N* (%)			0.234	0.889
I/II	13 (12.62)	19 (10.73)		
III	3 (2.91)	5 (2.82)		

### 3.1. Comparison of postoperative quality of life between the two groups

#### 3.1.1. QLQ-STO22 scale

After 1 year of follow-up, 2 patients in the U-RY group had recurrence and metastasis (1 case of liver metastasis and 1 case of anastomotic recurrence). One patient in the BII + Braun group had abdominal metastasis. Three patients in the U-RY group died (one with liver metastasis, one with coronary heart disease, and one with tumor recurrence) and two patients in the BII + Braun group died (one with coronary heart disease, and one with tumor recurrence).

According to the quality of life questionnaire 1 year after surgery, there were 100 cases in the U-RY group and 175 cases in the BII + Braun group.The QLQ-ST22 scores showed that compared to the BII + Braun group, the U-RY group had a lower pain score (8.5 ± 11.1 vs. 11.9 ± 9.7, *P* = 0.009) and reflux score (7.9 ± 8.5 vs. 11.0 ± 11.5, *P* = 0.012), and the difference was statistically significant at 1 year after surgery (*P* < 0.05). The U-RY group had a higher quality of life (Table 3).

**Table 3:** Comparison of QLQ-STO22 score items between the two groups in 1 year after surgery.

Group	Dysphagia	Pain	Reflux symptom	Eating restrictions	Having a dry mouth	Taste	Anxiety	Body image	Hair loss
U-RY group (*n* = 100)	6.6 ± 8.5	8.5 ± 11.1	7.9 ± 8.5	9.2 ± 9.7	7.0 ± 7.5	6.5 ± 6.9	8.6 ± 8.3	5.9 ± 6.4	7.3 ± 7.4
BII + Braun group (*n* = 175)	7.6 ± 9.6	11.9 ± 9.7	11.0 ± 11.5	10.4 ± 9.1	8.1 ± 8.3	7.7 ± 6.6	8.2 ± 9.1	6.7 ± 6.9	8.3 ± 8.6
T	0.843	2.627	2.526	1.076	1.033	1.399	0.349	0.974	1.027
*P*-value	0.400	0.009	0.012	0.283	0.303	0.163	0.727	0.331	0.305

#### 3.1.2. Endoscopic findings

One year after surgery, a total of 241 patients underwent endoscopic evaluation, including 92 cases in the U-RY group and 149 cases in the BII + Braun group. Compared to Billroth II with Braun group, the uncut Roux-en-Y group had significantly lower incidences of gastric stasis [16.3% (15/92) vs. 28.2% (42/149), *χ*^2^ = 4.448, *P* = 0.035], gastritis [13.0% (12/92) vs. 24.8% (37/149), *χ*^2^ = 4.880, *P* = 0.027] and bile reflux [2.2% (2/92) vs. 20.8% (11/149), *χ*^2^ = 16.707, *P* < 0.001], and the differences were statistically significant. There was no reflux esophagitis in U-RY group, but 3 cases in the BII + Braun group (2.0%). There was no significant difference between the two groups (*χ*^2^ = 1.876, *P* = 0.171). No recanalization was found in the U-RY obliterated afferent jejunal limb 1 year after surgery. However, recanalization occurred in 5 cases (4.9%) during the follow-up of 1–5 years after surgery. The results of this study suggest that the risk of recanalization of the proximal jejunal closure point of the input loop increases with the prolongation of postoperative time.

### 3.2. Comparison of postoperative survival status of the two groups

**Figure 5:**
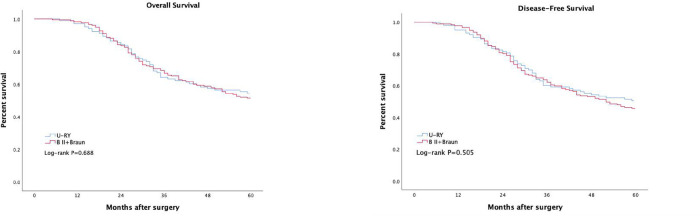
Comparison of long-term curative effect between two groups of patients. (**A**) Overall survival, (**B**) Disease-free survival.

There was no significant difference in overall survival (OS) and disease-free survival (DFS) between the U-RY group and the BII + Braun group (Figure 5). There was no significant difference in recurrence and metastasis between the U-RY group and the B II + Braun group (Table 4).

**Table 4:** Comparison of the long-term outcomes of the two groups.

Outcome	U-RY group (*N* = 103)	B II + Braun group (*N* = 177)	χ^2^	*P-*value
Status at the last follow-up, *N* (%)			0.324	0.569
Survival	56 (54.37%)	90 (50.85%)		
Dead	47 (45.63%)	87 (49.15%)		
Local recurrence, *N* (%)	10 (9.71%)	24 (13.56%)	0.905	0.341
Distant metastasis, *N* (%)	16 (15.53%)	28 (15.82%)	0.004	0.950
Liver metastasis, *N* (%)	9 (8.74%)	17 (9.60%)		
Lung metastasis, *N* (%)	7 (6.80%)	11 (6.21%)		

## 4. Discussion

Gastric cancer is a serious public health threat, and surgical treatment has become the first option (1). U-RY has been increasingly used in radical gastric cancer surgery, but it is still in the exploratory stage (5–7).

There were no significant differences between the two groups in operative time, intraoperative blood loss, postoperative complications, first exhaust time, time to liquid diet, and length of postoperative hospital stay (all *P* > 0.05); The results suggest that U-RY is safe and feasible for postoperative digestive tract reconstruction of distal gastric cancer. Postoperative complications are also an important index to evaluate the safety of operation. Postoperative complications not only prolong the length of stay, increase the cost, but also reduce trust between doctors and patients. The total incidence of postoperative complications in the U-RY group and BII + Braun group was 15.53% (16/103) and 14.12% (25/177), respectively, and there was no statistical difference (*P* > 0.05). U-RY reconstruction is a modification of BII + Braun anastomosis, which has an additional staple line across the afferent limb to block bile reflux. In terms of quality of life 1 year after surgery, the score of pain and reflux symptoms in the U-RY group was better than that in the BII + Braun group, indicating that it could significantly improve the **quality of life** of patients after surgery (8, 9). Quality of life has always been regarded as one of the important indicators for measuring the quality of life of cancer patients (10).

In terms of postoperative gastric reflux symptoms, many studies have shown that U-RY can prevent pancreatic juice and bile reflux, reduce the incidence of Roux-en-Y stasis syndrome (RSS), and improve the quality of life of patients after surgery (11, 12). Pain is a subjective feeling, which is generally thought to be affected by postoperative abdominal adhesion, the incidence and severity of reflux symptoms, cholelithiasis, and other factors. In terms of pain score, the result of U-RY group is better than that of BII + Braun, which may benefit from the closure point of U-RY anastomosis.The symptoms of related residual gastritis caused by reflux were significantly alleviated.Reducing reflux and pain is helpful to improve the overall health status of patients in U-RY group after surgery.

The results of this study showed that there was no recanalization of the proximal jejunal closure point of the input loop within 1 year after U-RY, but recanalization occurred in 5 (4.9%) cases during follow-up 1–5 years after operation. The results suggest that with the extension of postoperative time, the risk of recanalization of the proximal jejunal closure point of the input loop will increase, resulting in alkaline reflux to the residual stomach and inflammatory lesions of the esophagus (13, 14). Since the application of U-RY anastomosis in the treatment of distal gastric cancer, the recanalization rate of the closed point has gradually decreased, not only because of the continuous development of closed materials and medical instruments, but also the distance between the closed point of the input loop and gastrointestinal anastomosis, which is also the key point to prevent recanalization and improve the quality of life. At the same time, we found that the length of the jejunal stump of the input loop was no longer surgically designed 3–5 cm in the digestive tract imaging, and the distance of the jejunal stump had changed to 4–8 cm after 5 years. Therefore, we speculate that the food deposited in the blind loop of the jejunum is pushed and hits the closing point, which leads to adaptive elongation of the intestinal stump. With the increase in the length of the intestinal stump, according to the relationship between pressure and height or length, the pressure on the closing point gradually increases, which explains why the recanalization rate will gradually increase with the extension of time.Therefore, as long as there is a distance between the closing point and gastrointestinal anastomosis, with the extension of time, it will be constantly squeezed by food, resulting in intestinal adaptation elongation, and the recanalization rate will increase. Therefore, we suggest that the shorter the length of the jejunal stump, the better. At the same time, relevant clinical studies found that the incidence of postoperative intestinal obstruction in the U-RY group was significantly higher than that in the Roux-en-Y anastomosis group (*P* = 0.027). In the U-RY group, postoperative intestinal obstruction occurred in 6 patients (17.6%), all within half a year after surgery (15). To analyze the reason, the researchers chose the closing point at the distance from the gastrojejunostomy to the 5 cm, and a “pocket” device may be formed between the gastrointestinal anastomosis and the closure point, which may lead to food confinement between the closure point and the gastrojejunal anastomosis, leading to emptying disorders or intestinal obstruction. After that, we improved the procedure and chose the closure point at 2–3 cm near the gastrojunostomy. Follow-up results showed that the quality of life of patients who underwent U-RY anastomosis at a later stage was significantly improved. However, the follow-up time and cases in the study are limited, so the problem of recanalization needs further follow up.

In our study, there was no difference in the incidence of postoperative abdominal infection and tumor recurrence between the two groups. Related clinical studies have shown that intraoperative lymph node dissection also has a significant impact on patient prognosis (16). In this study, there was no statistical significance in the number of lymph node dissection between the two groups. There was no significant difference in tumor recurrence, metastasis and long-term survival between the two groups. Adequate preoperative preparation, prophylactic use of antibiotics, use of aseptic gloves, adequate lymph node dissection, and strict adherence to aseptic and tumor-free principles ensure the safety of U-RY surgery. For gastric cancer patients undergoing surgery, the long-term effect of surgery has a direct impact on patient survival. There was no significant difference in recurrence, distant metastasis, OS, and DFS between the two groups. These results suggest that the long-term effect of U-RY on gastric cancer is the same as that of BII + Braun. However, U-RY digestive tract reconstruction is limited by mesenteric length, and there may be a problem of excessive anastomotic tension. We strictly evaluate the patient's condition and surgical indications to avoid surgical failure.

The author acknowledges that this study has some limitations. First of all, this study is a single-center retrospective study, the number of cases is relatively small, the conclusion of evidence-based medical evidence is still low, the results still need large samples, multi-center randomized controlled trials to further verify. Secondly, the QLQ-STO22 scale data are obtained by questionnaire, and the results may have a certain degree of deviation due to the influence of patients' emotions, cultural background and education level.

## Data Availability

The raw data supporting the conclusions of this article will be made available by the authors, without undue reservation.
